# Draft genome sequencing of multidrug-resistant *Klebsiella pneumoniae* from neonatal bloodstream infections at SRHU, Dehradun, India

**DOI:** 10.1128/mra.00619-25

**Published:** 2026-01-22

**Authors:** Indrani Sarkar, Arpana Singh, Uttam Kumar Nath, Barnali Kakati, Saugata Hazra

**Affiliations:** 1 Department of Medical Oncology and Haematology, AIIMS Rishikesh 442339, Rishikesh, Uttarakhand, India; 2 Department of Biosciences and Bioengineering, Indian Institute of Technology-Roorkee 30112, Roorkee, Uttarakhand, India; 3 Centre for Nanotechnology, Indian Institute of Technology-Roorkee 30112, Roorkee, Uttarakhand, India; 4 Department of Microbiology, Swami Rama Himalayan University 422223, Dehradun, Uttarakhand, India; University of Pittsburgh School of Medicine, Pittsburgh, Pennsylvania, USA

**Keywords:** multidrug resistance, *Klebsiella pneumoniae*, whole-genome sequencing, antibiotics, sepsis

## Abstract

*Klebsiella pneumoniae,* a gram-negative opportunistic pathogen, causes pneumonia, blood infection, meningitis along with several wound and surgical site infections and has become a leading cause of multidrug-resistant nosocomial infection. Here we present the whole-genome sequences for five *K. pneumoniae* strains isolated from clinical samples, Dehradun, India.

## ANNOUNCEMENT

*Klebsiella pneumoniae* is a gram-negative, non-motile bacillus present in skin, mouth, and gastrointestinal tract of all humans (1). Here, we are reporting the five multidrug resistance (MDR) Indian *K. pneumoniae* genome sequences, which were isolated from blood samples from neonates with sepsis (IEC No. 02/IEC/EM/2024). Blood samples were collected in pediatric blood culture bottles of BacT/Alert system and sent to the bacteriology laboratory in the Department of Microbiology. The blood culture bottles were incubated at 37°C in an automated blood culture system as per the standard protocol. Once they flagged positive for microbial growth, direct microscopy using Gram staining was done to observe gram-negative short, plump capsulated bacilli, followed by subculture on 5% sheep blood agar and MacConkey agar. The plates were incubated aerobically at 37°C for 18–24 h. On MA, lactose-fermenting, mucoid colonies indicative of *Klebsiella* species were observed. Antimicrobial susceptibility testing was performed and interpreted as per standard guidelines (2). A single well-isolated colony was inoculated into nutrient broth and incubated at 37°C for 16–18 h, and DNA isolation was performed using the Xploregen DNA isolation kit. DNA was quantified with the QUBIT 3 Fluorometer and Qiagen dsDNA HS Dye for exact quantification. The Kapa DNA library preparation kit was used for library preparation. Whole-genome sequencing was performed on the Illumina Novaseq 6000 platform using paired-end (151 read length) configuration. Trimgalore v0.6.8 was used for adapter trimming. FastQC v0.12.1 (3) was used to assess quality. The raw reads were assembled with Unicycler v0.5.0 (4), and the resultant sequences were processed with MeduSa 2.0 (5) to ensure high-quality results. BUSCO 5.8.2 (6) was used to calculate the completeness. Genome annotation was performed by NCBI-PGAP (7). ResFinder 4.7.2 (8) and PlasmidFinder 2.1 (9) were used to identify the antimicrobial resistance genes and the plasmids from the secondary assembly data. All software and web servers were used with default parameters. Table 1 provides the details of genome statistics of the newly sequenced genomes.

**TABLE 1:** Overall statistics of the five sequenced genomes

Genome name	No. of reads	Genome completeness	No. of contigs	Genome coverage	*N*_50_	GC%	Predicted antibiotic resistance	Plasmid types
HIMS_HL_K002	5,571,571	98.7	26	148.53×	5,132,138	54	Cefepime, amoxicillin + clavulanic acid, ampicillin + clavulanic acid, ertapenem, imipenem, meropenem, and fosfomycin	IncFIB, IncFII, and IncR
HIMS_HL_K003	5,587,282	98.7	16	187.57×	187,208	54.5	Amoxicillin + clavulanic acid, ampicillin + clavulanic acid, cephalothin, sulfamethoxazole, and fosfomycin	IncFIA, IncFIB, and repB
HIMS_HL_K009	5,736,184	98.7	18	211.57×	314,957	54	Ticarcillin +clavulanic acid, amoxicillin + clavulanic acid, ampicillin + clavulanic acid, chloramphenicol, fosfomycin, nalidixic acid, and trimethoprim	Col440II, IncFIA, IncFIB, IncFII, IncX3, and repA
HIMS_HL_K010	5,607,347	98.7	21	187.02×	296,973	53	Chloramphenicol, fosfomycin, ciprofloxacin, nalidixic acid, and trimethoprim	Col440II, IncFIA, IncFIB, IncFII, IncR, and IncX3
HIMS_HL_K016	5,715,641	98.7	22	159.98×	301,136	57	Cefotaxime, ceftazidime, cefixime, cefoxitin, amoxicillin, amoxicillin + clavulanic acid, ampicillin, cephalothin, chloramphenicol, fosfomycin, and nalidixic acid	Col440I, ColKP3, IncFIB, IncFII, IncQ1, and IncR

The study found numerous antibiotic resistance genes in the sequenced strains resulting in broad-spectrum antibiotic resistance. HIMS_HL_K002 possesses blaTEM, blaNDM, blaCTX-M, blaSHV, and fosA providing resistance against a broad range of beta-lactams along with Fosfomycin. blaDHA, blaSHV, fosA, and sul-1 were identified in HIMS_HL_K003. blaSHV and OqxB were identified in HIMS_HL_K009. OqxB and CatA were obtained in HIMS_HL_K010, and blaTEM, blaOXA, blaNDM, blaCTX-M, blaSHV, Sul-2, fosA6, and Oqx-B were identified in HIMS_HL_K016. Presence of these genes marked the clinical significance of these isolates as MDR pathogens.

## Data Availability

These genomes are available under the BioProject ID PRJNA1264554. The GenBank accession numbers and BioSample numbers are HIMS_HL_K002 (JBOCYR000000000, SAMN48564312), HIMS_HL_K003 (JBOCYQ000000000, SAMN48564313), HIMS_HL_K009 (JBOCYK000000000, SAMN48564319), HIMS_HL_K010 (JBOCYJ000000000, SAMN48564320), and HIMS_HL_K016 (JBOCYE000000000, SAMN48564325).
